# Endovascular iliac vein reconstruction through an obstructive pelvic nodal recurrence of urothelial carcinoma

**DOI:** 10.1186/s42155-018-0024-2

**Published:** 2018-08-30

**Authors:** Bedros Taslakian, Varshaa Koneru, Akhilesh K. Sista

**Affiliations:** 1 Department of Radiology, Vascular and Interventional Radiology Section, NYU Langone Medical Center, 550 1st Avenue, 2nd Floor, New York, NY 10016 USA; 2 Department of Medicine, Baton Rouge General Medical Center, Baton Rouge, LA USA; 3 Department of Radiology, Division of Vascular and Interventional Radiology (VIR), NYU Langone Medical Center, 660 1st Avenue, 3nd Floor, New York, NY 10016 USA

**Keywords:** Sharp recanalization, Deep venous thrombosis, Venous compression, Endovenous intervention

## Abstract

**Background:**

Chronic venous occlusion is common particularly in cancer patient due to hypercoagulate state associated with venous compression. Treatment options include endovascular management with venoplasty and stenting. Recanalization can be challenging in patients with complete venous occlusion secondary to significant external compression by a mass.

**Case presentation:**

We report a case of a 73-year-old man with a history of bladder and prostate cancer who presented with worsening right leg edema and pain due to deep venous thrombosis secondary to a retroperitoneal mass. Management was sharp recanalization, venoplasty and stenting.

**Conclusion:**

Endovascular intervention of chronic venous occlusion is technically challenging and time consuming. Sharp venous recanalization is feasible and safe in patients who failed standard recanalization procedures. We present a case of cancer-related obstruction of the right iliac veins and acute thrombosis of the femoral vein with symptomatic lower limb swelling relieved by sharp recanalization through the tumor mass.

## Background

Deep venous thrombosis (DVT) in cancer patients causes significant morbidity and affects the quality of life (Blom et al. 2005; Delis et al. 2004). Acute DVT can be attributed to hypercoagulable state, compression, or both (Blom et al. 2005). Percutaneous endovascular intervention with or without thrombolysis may be performed to improve symptoms in acute DVT (Maleux et al. 2016; Sullivan GJ et al. 2015; Neglén et al. 2007; Murphy et al. 2017). When the venous inflow or outflow is compromised due to external compression or intra-luminal stenosis, thrombolysis alone may not be effective and the recanalized veins can re-thrombose.

Chronic venous occlusions that cannot be crossed with standard wire and catheter combinations are technically challenging. Lesion crossing attempts fail in approximately 5–24% of the cases of long-standing peripheral occlusions (Raju and Neglén 2009; Raju 2013; Raju 2015; Criado et al. 1994). When standard catheter and guidewire techniques do not permit connection of two patent lumens, sharp recanalization might be an option (Honnef et al. 2005; Athreya et al. 2009; Farrell et al. 1999; Dou et al. 2016). We describe a case of cancer-related iliofemoral venous occlusion and secondary acute DVT causing painful lower limb swelling which was managed by percutaneous extravascular venous bypass through an encasing nodal tumor using sharp recanalization, after the failure of conventional techniques. While it is not recommended to routinely pass through tumor because of the theoretical risk of tumor seeding and bleeding, an exception was made here because the patient was suffering from severe symptoms and the goal of care was palliation.

## Case presentation

A 73-year-old man with past medical history of stage IV bladder and prostate cancer, status post cystoprostatectomy with ileal conduit, left orchiectomy, and subsequent left radical nephrectomy (for recurrent left hydronephrosis and pyelonephritis), presented with worsening right leg edema and pain for 2–3 weeks which confined him to bedrest. On examination, there was severe pitting edema of the right leg associated with erythema and warmth.

**Fig. 1:**
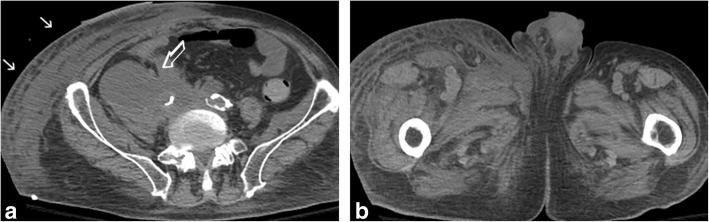
Non-contrast CT images through (**a**) the lower pelvis and (**b**) upper thighs demonstrating a large obstructing right pelvic tumor (open arrow), significant right-sided subcutaneous edema (arrows), and marked limb asymmetry due to right limb edema

Duplex ultrasound examination of the lower extremities revealed a nonocclusive thrombus extending from the right external iliac to the central (cranial) segment of the femoral vein and an occlusive thrombus in the central segment of the deep femoral (profunda femoris) vein. A non-contrast CT scan of the abdomen and pelvis revealed an increase in the size of a pelvic retroperitoneal nodal mass inseparable from the right common iliac artery and right ureter, with no clear visualization of the right iliac venous system (Fig. 1). The patient was placed on therapeutic low molecular weight heparin in preparation for right lower extremity catheter-directed thrombolysis and stenting.

**Fig. 2:**
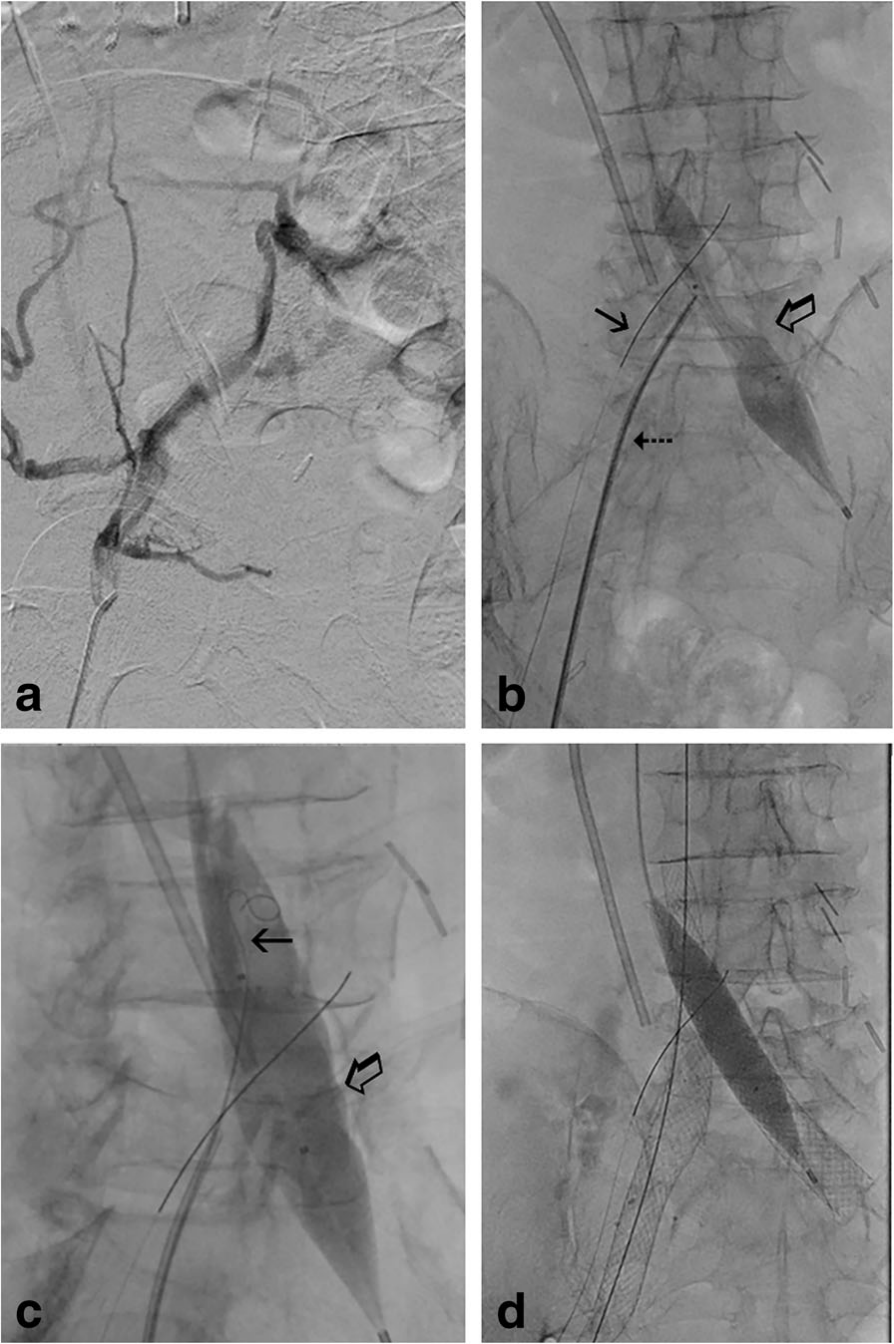
**a** Venography through the right popliteal venous access demonstrates complete occlusion of the native right iliac veins and extensive collateralization. **b** the tip of a 0.018″ wire inserted though the common iliac artery ends in the lower aorta (solid arrow). A transjugular metal cannula with a coaxial 21-gauge Chiba needle (dotted arrow) is inserted through the right common femoral vein and is about to enter the IVC which contains a semi-inflated balloon inserted through the internal jugular vein (open arrow). **c** The tip of a 0.018-in. wire (arrow) inserted through the created channel now ends in the IVC alongside the inflated balloon (open arrow). **d** Kissing stents were placed through the right femoral vein and internal jugular vein access

Pre-procedural ultrasound examination of the access site confirmed findings seen on lower extremity Doppler examination and revealed extension of the venous thrombosis into the femoral and deep femoral vein precluding femoral vein access. The patient was then placed in prone position under general anesthesia and the right popliteal vein was accessed. Venography showed extensive thrombosis and strictures of the right common iliac, right external iliac, and right femoral veins with collateralization (Fig. 2a). Pharmacomechanical thrombolysis using the Trellis Thrombectomy System (Formerly Covidien, Boston, MA, now discontinued) was performed in the right femoral vein through the popliteal vein access to clear the acute thrombus. Repeat venography showed resolution of the acute DVT in the right femoral and external iliac veins with minimal residual stenosis. There was however no identifiable connection between the right common iliac vein and the inferior vena cava (IVC). Unsuccessful attempts were made using multiple wire/catheter combinations to recanalize the right common iliac vein. Initial attempts to cross the obstruction using an angled catheter in combination with soft and stiff hydrophilic wires were unsuccessful. Furthermore, attempts were made using a crossing Rubicon Support Catheter (Boston Scientific, Marlborough MA) in combination with hydrophilic wires and the stiff (back) end of an Amplatz wire. However, there was inadequate support with this combination. The left popliteal vein was then accessed using a micro puncture set and venography demonstrated a narrow, but patent left common iliac vein and patent IVC without identifiable inflow from the right common iliac vein.

The patient was subsequently turned supine. A wire was advanced through a right common femoral artery access into the lower aorta to provide a visual safeguard for preventing arterial injury during sharp venous recanalization. An 18 mm Atlas balloon (Bard, Murray Hill, NJ) placed through a right internal jugular access was inflated across the IVC/left common iliac vein confluence to provide a central target. A 10 French right femoral venous sheath was inserted and the metallic stiffening cannula/catheter combination from a Rösch-Uchida Transjugular Liver Access Set was advanced into the peripheral (caudal) segment of the right common iliac vein stump. The metal cannula was then progressively advanced towards the target balloon in the lower IVC. A 21-gauge Chiba needle was advanced through the cannula (Fig. 2b). The needle and a 0.018-in. guidewire were used to create a channel through the encasing tumor into the caudal segment of the IVC with one pass (Fig. 2c).

**Fig. 3:**
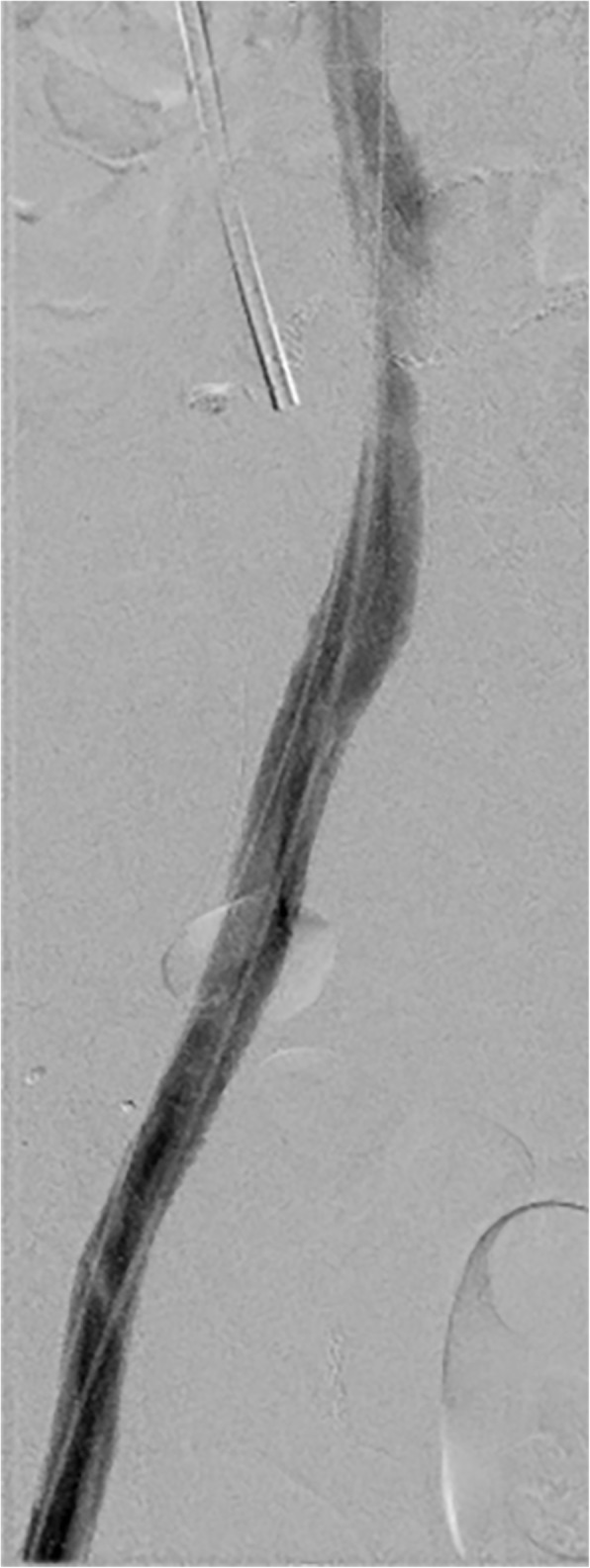
Completion venography shows widely patent right iliac vein stents with free contrast flowing into the IVC

After gaining access into the patent IVC, balloon angioplasty was performed and self-expanding kissing [16 mm diameter × 90 mm length] Wallstents (Boston Scientific, Marlborough MA) were placed in both common iliac veins. Three additional overlapping stents [one 16 mm × 90 mm Wallstent; two 14 mm × 60 mm S.M.A.R.T stents (Cordes, Fremont, CA)] were placed from the right common iliac to the right common femoral vein followed by balloon angioplasty (Fig. 2d). Initial post-stenting venography demonstrated thrombosis of the central stents, likely due to inadequate inflow. Pharmacomechanical thrombolysis using the Trellis Thrombectomy System was performed to successfully remove the acute thrombus from the central stents. The stents were extended below the femoral head to ensure adequate inflow using an additional 10 mm × 40 mm EV3 Protégé (Medtronic, Minneapolis MN) stent. Completion venography demonstrated widely patent stents with contrast flowing into the IVC (Fig. 3). The catheters were removed, and hemostasis achieved. Over the next several days, there was significant improvement of the pain and swelling and the patient was discharged to hospice care. The patient passed away after 2 months due to progression of the metastatic disease.

## Conclusions

Lower extremity deep venous disease can be extremely debilitating for cancer patients (Blom et al. 2005; Delis et al. 2004). While more data is needed, preliminary results suggest that this population can obtain significant palliation from endovenous interventions such as thrombolysis, angioplasty, and stenting (Neglén et al. 2007; Murphy et al. 2017). Most lesions are readily crossed with standard guidewires and catheters. In select cases, when standard techniques fail, novel creation of venous flow through sharp recanalization technique as described above may be useful.
